# Nephron-Sparing Surgery for Adult Xp11.2 Translocation Renal Cell Carcinoma at Clinical T1 Stage: A Multicenter Study in China

**DOI:** 10.1245/s10434-020-08813-y

**Published:** 2020-07-06

**Authors:** Ning Liu, Feng Qu, Qiancheng Shi, Wenyuan Zhuang, Wenliang Ma, Zhenhao Yang, Jing Sun, Wei Xu, Lihua Zhang, Ruipeng Jia, Linfeng Xu, Xiaozhi Zhao, Xiaogong Li, Gutian Zhang, Hongqian Guo, Dongmei Li, Weidong Gan

**Affiliations:** 1 Department of Urology, Nanjing Drum Tower Hospital, The Affiliated Hospital of Nanjing University Medical School, Nanjing, Jiangsu Province People’s Republic of China; 2 Department of Urology, Drum Tower Clinical Medical School of Nanjing Medical University, Nanjing, China; 3 Department of Urology, Nanjing Drum Tower Hospital Clinical College of Traditional Chinese and Western Medicine, Nanjing University of Chinese Medicine, Nanjing, China; 4 Department of Oncology, Jiangsu Province Hospital, The First Affiliated Hospital of Nanjing Medical University, Nanjing, China; 5 Department of Pathology, Jiangsu Cancer Hospital, The Affiliated Cancer Hospital of Nanjing Medical University, Nanjing, China; 6 Department of Pathology, Zhongda Hospital Southeast University, Nanjing, China; 7 Department of Urology, Nanjing First Hospital, The Affiliated Nanjing Hospital of Nanjing Medical University, Nanjing, China; 8 Immunology and Reproduction Biology Laboratory and State Key Laboratory of Analytical Chemistry for Life Science, Medical School, Nanjing University, Nanjing, China; 9 Jiangsu Key Laboratory of Molecular Medicine, Nanjing University, Nanjing, China

## Abstract

**Purpose:**

To evaluate the oncologic efficacy and feasibility of nephron-sparing surgery (NSS) in adult Xp11.2 translocation renal cell carcinoma (RCC).

**Patients and Methods:**

Seventy patients with Xp11.2 translocation RCC and 273 with conventional RCC from five institutions in Nanjing were retrospectively studied. All patients were older than 18 years and were categorized into clinical T1 (cT1) stage using preoperative imaging. Using the preoperative imaging and electronic medical records, anatomical and pathological features were collected and analyzed.

**Results:**

Among patients with Xp11.2 translocation RCC, 18/36 (50.0%) with cT1a and 12/34 (35.3%) with cT1b tumors underwent NSS. The respective proportions in the conventional RCC group were 121/145 (83.4%) and 93/128 (72.7%). Among cT1a tumors, the Xp11.2 translocation RCCs tended to be adjacent to the collecting system, sinus, and axial renal midline compared with conventional RCCs. Patients with Xp11.2 translocation RCCs who underwent NSS had comparable progression-free survival (PFS) and overall survival to radical nephrectomy (RN) patients (*P *> 0.05). Among cT1b tumors, surgical margin positivity and pelvicalyceal, vascular, and region lymphatic involvement were more likely to occur in the Xp11.2 translocation RCCs (*P *< 0.05). Patients with Xp11.2 translocation RCC who underwent RN had a more favorable PFS than those who underwent NSS (*P *= 0.048). However, multivariate analysis of PFS did not identify surgical method as a risk factor (*P *= 0.089).

**Conclusions:**

Among adults with Xp11.2 translocation RCC, NSS can be an alternative for patients with cT1a tumor but should be performed with more deliberation in patients with cT1b tumors.

**Electronic supplementary material:**

The online version of this article (10.1245/s10434-020-08813-y) contains supplementary material, which is available to authorized users.

Xp11.2 translocation renal cell carcinoma (RCC) is a rare type of RCC associated with balanced translocation of transcription factor E3 (*TFE3*) and other fusion partners. The World Health Organization recognized Xp11.2 translocation RCC as a distinctive RCC entity in 2004^1^ and reclassified it into microphthalmia transcription factor family translocation RCCs in 2016.^2^ Xp11.2 translocation RCC shows a more invasive course and more aggressive biological behavior than non-Xp11.2 translocation RCC,^3^,^4^ although Xp11 translocation RCC and clear cell RCC (ccRCC) have similar clinical courses.^5^ Up to half of patients with Xp11.2 translocation RCC present with regional progress or metastasis.^6^,^7^ Currently, complete excision is the most effective therapy for local Xp11.2 translocation RCC.

Radical nephrectomy (RN) remains the classical therapy with a reliable oncologic outcome for local RCC. By complete resection of the primary tumor and maximal preservation of the tumor-bearing kidney, nephron-sparing surgery (NSS) could realize oncologic efficacy as well as reduction in complications.^8^ Current guidelines recommend elective NSS as the standard surgical treatment for T1a renal tumors and favor NSS over RN for T1b tumors when technically feasible.^9^ However, studies have reported varied oncologic outcomes of NSS in diverse histological subtypes.^10^,^11^ Systematic reports on oncologic outcomes of Xp11.2 translocation RCC after NSS are still lacking. Due to the hysteresis of pathological diagnosis, an increasing number of Xp11.2 translocation RCCs at clinical T1 (cT1) stage underwent NSS, the same surgical strategy as that for conventional RCC. Considering the aggressive biological behavior of Xp11.2 translocation RCC, we designed a multicenter study to investigate the feasibility of NSS for Xp11.2 translocation RCC based on the most comprehensive clinical data.

## Patients and Methods

### Study Design and Patient Selection

**Fig. 1:**
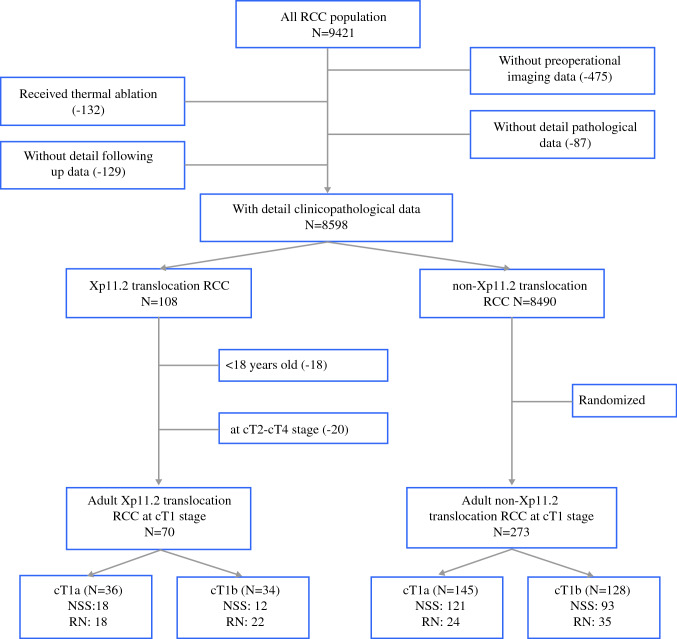
Study design and patient selection

The present retrospective study was approved by the institutional review board and performed in accordance with the ethical standards established by the five institutions. Altogether, 8598 cases of RCC with detailed clinicopathological data were reported between January 2007 and December 2019. Among these, 108 cases were eventually diagnosed as Xp11.2 translocation RCC by immunohistochemical staining^12^ and fluorescence in situ hybridization at five institutions.^13^,^14^ Clinicopathological data of patients with conventional RCC from these institutions during the same period were also randomly collected and used as controls (Fig. 1). Eligibility criteria for inclusion in the study were patients older than 18 years, with a solitary tumor at cT1 stage, a normal contralateral kidney, integrated imaging data, accurate pathological diagnosis, and a satisfactory follow-up. cT1 tumor was defined as a tumor ≤ 7 cm in the greatest dimension and limited to the kidney based on preoperative computed tomography (CT) imaging data. After verification of the eligibility criteria, 343 patients with cT1 tumors were centrally randomized and retrospectively studied. These included 70 patients with Xp11.2 translocation RCC and 273 with conventional RCC (Supplementary Material 1). The latter group comprised 167 patients with ccRCC, 63 with papillary RCC (pRCC), 30 with chromophobe RCC (chRCC), and 13 with clear cell papillary RCC.

### Assessment of Variables

Clinical data including epidemiological features (sex and age), clinical manifestations, preoperative CT imaging (tumor size, laterality, and RENAL score), surgical methods (NSS or RN), clinical outcomes, and follow-up information were collected from the medical records. Detailed pathological features are an important reflection of tumor biological behavior. Information about pathological features including necrosis, sarcomatous, venous cancer thrombus, lymphatic metastasis, and other local invasion factors was also collected. Cases that underwent partial nephrectomy or tumor enucleation were included in the NSS group, while cases with complete removal of the affected kidney were included in the RN group.

According to the American Joint Committee on Cancer staging criteria (eighth edition, 2017), primary tumors were divided into the cT1a (≤ 4 cm in the greatest dimension, clinically limited to the kidney) group and the cT1b (> 4 cm and ≤ 7 cm in the greatest dimension, clinically limited to the kidney) group. Anatomical features are important factors that influence therapeutic strategies. The RENAL scores (Kutikov and Uzzo),^15^ which provide a standard way of describing anatomical features, were evaluated in consensus by a radiologist (Jian He) and a urologist (Feng Qu) based on patients’ preoperative CT imaging findings. The imaging data were reviewed on a picture archiving and communication system workstation (GE AW4.3; GE Healthcare, Chicago, IL). Survival data including progression-free survival (PFS) and overall survival (OS) were obtained from electronic medical records. PFS was defined as the time from the initiation of the surgery to the date of disease progression or censoring at the time of the last follow-up. OS was defined as the time interval between the date of the surgery and the date of death or the last follow-up.

### Statistical Analysis

Patient baseline characteristics were analyzed using descriptive statistics. Continuous and categorical variables were analyzed using independent *t* test and Chi squared test, respectively. Univariate and multivariate Cox regression analyses were used to evaluate the predictive role of all factors for survival. PFS and OS curves were obtained by Kaplan–Meier analysis, and survival comparisons were performed using log-rank test. Statistical analyses were performed using IBM SPSS Statistics version 23.0 (IBM Corp., Armonk, NY), and the level of statistical significance was set at *P *< 0.05.

## Results

Xp11.2 translocation RCCs accounted for 1.26% of all RCC cases. The mean age at onset of Xp11.2 translocation RCC was 32.2 ± 17.0 years, which was significantly lower than that of conventional RCC. There was a slight male predominance in the conventional RCC group, but this predominance was reversed in the Xp11.2 translocation RCC group (*P *< 0.001). Detailed clinicopathological characteristics of the 343 cases are summarized in Table 1. Among cT1a tumors, 18/36 (50.0%) cases of Xp11.2 translocation RCC and 121/145 (83.4%) cases of conventional RCC underwent NSS. Among cT1b tumors, NSS was performed in 12/34 (35.3%) cases of Xp11.2 translocation RCC and in 93/128 (72.7%) cases of conventional RCC. Thus, Xp11.2 translocation RCCs at cT1a stage were more likely to undergo RN than conventional RCCs (*P *< 0.05).

**Table 1:** Descriptive characteristics of Xp11.2 translocation and conventional renal cell carcinoma at clinical T1 stage

Variable (%)	Xp11.2 translocation RCC (*N* = 70)	Conventional RCC (*N* = 273)	*P*-value
Age, years (mean ± SD)	32.2 ± 17.0	56.5 ± 11.9	< 0.001
Gender			< 0.001
Male	30 (42.9%)	184 (67.4%)	
Female	40 (57.1%)	89 (32.6%)	
Side			0.930
Left	36 (51.4%)	142 (52.0%)	
Right	34 (48.6%)	131 (48.0%)	
Tumor diameter			0.801
cT1a	36 (51.4%)	145 (53.1%)	
cT1b	34 (48.6%)	128 (46.9%)	
Tumor size, cm (mean ± SD)	4.4 ± 1.3	4.2 ± 1.5	0.267
Fuhrman grade			0.002
Grade I–II	31 (44.3%)	176 (64.5%)	
Grade III–IV	39 (55.7%)	97 (35.5%)	
Surgery			< 0.001
NSS	30 (42.9%)	214 (78.4%)	
RN	40 (57.1%)	59 (21.6%)	

*NSS* nephron-sparing surgery, *RN* radical nephrectomy

The RENAL scores of patients are presented in Table 2. Regardless of the stage (cT1a or cT1b), Xp11.2 translocation RCCs were located closer to the collecting system or the sinus than conventional RCCs. Among cT1a tumors, Xp11.2 translocation RCCs were more likely to cross the axial renal midline or to be located entirely between the polar lines. Among cT1b tumors, Xp11.2 translocation RCCs showed a higher rate of endophytic growth. These features collectively contributed to the higher total RENAL scores of Xp11.2 translocation RCC.

**Table 2:** Anatomical features of Xp11.2 translocation and conventional renal cell carcinoma at clinical T1 stage

Anatomic features	Clinical T1a stage			Clinical T1b stage		
	Xp11.2 translocation RCC (36 cases)	Conventional RCC (145 cases)	*P*-value	Xp11.2 translocation RCC (34 cases)	Conventional RCC (128 cases)	*P*-value
Radius, cm (diameter, mean ± SD)	3.27 ± 0.60	3.08 ± 0.79	0.304	5.58 ± 0.74	5.56 ± 0.86	0.919
Exophytic/endophytic properties			0.117			0.003
≥ 50%	15 (41.7%)	76 (52.4%)		12 (35.3%)	70 (54.7%)	
< 50%	6 (16.7%)	30 (20.7%)		8 (23.5%)	39 (30.5%)	
Entirely endophytic	15 (41.7%)	39 (26.9%)		14 (41.2%)	19 (14.8%)	
Nearness of tumor to collecting system or sinus (mm)			0.009			< 0.001
> 7	5 (13.9%)	43 (29.7%)		2 (5.9%)	28 (21.9%)	
> 4 but < 7	9 (25.0%)	48 (33.1%)		3 (8.8%)	45 (35.2%)	
< 4	22 (61.1%)	54 (37.2%)		29 (85.3%)	55 (43.0%)	
Anterior or posterior or X			0.309			0.083
Anterior	18 (50.0%)	60 (41.4%)		17 (50.0%)	50 (39.1%)	
Posterior	15 (41.7%)	67 (46.2%)		13 (38.2%)	43 (33.6%)	
X	3 (8.3%)	18 (12.4%)		4 (11.8%)	35 (27.3%)	
Location relative to the polar lines			0.001			0.293
Entirely above upper or below lower polar line	8 (22.2%)	56 (38.6%)		4 (11.8%)	36 (28.1%)	
Lesion crosses polar line	4 (11.1%)	45 (31.0%)		12 (35.3%)	25 (19.5%)	
> 50% mass across polar line or mass crosses axial renal midline or is entirely between polar lines	24 (66.7%)	44 (30.3%)		18 (52.9%)	67 (52.3%)	
Total RNEAL score (mean ± SD)	7.92 ± 2.03	6.05 ± 1.73	0.003	9.26 ± 2.03	7.15 ± 1.73	0.012

Xp11.2 translocation RCCs were more likely to show necrosis (Table 3). The incidence rate of undefined border and advanced Fuhrman grade was significantly higher in Xp11.2 translocation RCCs at cT1a stage than that in conventional RCCs. Moreover, surgical margin positivity, pelvicalyceal invasion, vascular invasion, and lymphatic metastasis were more common in the Xp11.2 translocation RCCs in the cT1b group.

**Table 3:** Pathological features of Xp11.2 translocation and conventional renal cell carcinoma at clinical T1 stage

Pathological feature	Clinical T1a stage			Clinical T1b stage		
	Xp11.2 translocation RCC (36 cases)	Conventional RCC (145 cases)	*P*-value	Xp11.2 translocation RCC (34 cases)	Conventional RCC (128 cases)	*P*-value
Tumor size, cm (mean ± SD)	3.27 ± 0.60	3.08 ± 0.79	0.304	5.58 ± 0.74	5.56 ± 0.86	0.919
Necrosis	8/36 (22.2%)	12/145 (8.3%)	0.036	9/34 (26.5%)	66/128 (51.6%)	0.009
Sarcomatous	2/36 (5.6%)	6/145 (4.1%)	0.711	2/34 (5.9%)	18/128 (14.1%)	0.319
Renal capsule			0.072			0.543
Uninvolved	9/36 (25.0%)	66/145 (45.5%)		5/34 (14.7%)	15/128 (11.7%)	
Involved	18/36 (50.0%)	54/145 (37.2%)		22/34 (64.7%)	95/128 (74.2%)	
Breakthrough	9/36 (25.0%)	25/145 (17.2%)		7/34 (20.6%)	18/128 (14.1%)	
Undefined border	13/36 (36.1%)	18/145 (12.4%)	0.001	10/34 (29.4%)	44/128 (34.4%)	0.585
Surgical margin positivity	1/18 (5.6%)	5/121 (4.1%)	0.782	4/12 (33.3%)	8/93 (8.6%)	0.040
Adrenal invasion	0/4 (0%)	0/1 (0%)	–	1/8 (12.5%)	0/8 (0%)	0.228
Pelvicalyceal invasion	5/18 (27.8%)	2/24 (8.3%)	0.094	11/22 (50.0%)	2/35 (5.7%)	< 0.001
Nerve invasion	0/18 (0%)	0/24 (0%)	–	2/22 (9.1%)	1/35 (2.9%)	0.313
Vascular invasion	1/18 (5.6%)	0/24 (0%)	0.189	11/22 (50.0%)	7/35 (20.0%)	0.018
Venous cancer thrombus	0/18 (0%)	3/24 (12.5%)	0.060	4/22 (18.2%)	4/35 (11.4%)	0.480
Renal sinus invasion	1/18 (5.6%)	2/24 (8.3%)	0.726	2/22 (9.1%)	4/35 (11.4%)	0.778
Lymphatic metastasis	0/5 (0%)	0/3 (0%)	–	6/11 (54.5%)	1/9 (11.1%)	0.035
Fuhrman grade			< 0.001			0.969
I + II	16/36 (44.4%)	120/145 (82.8%).		15/34 (44.1%)	56/128 (43.8%)	
III + IV	20/36 (55.6%)	25/145 (17.2%)		19/34 (55.9%)	72/128 (56.3%)	

**Fig. 2:**
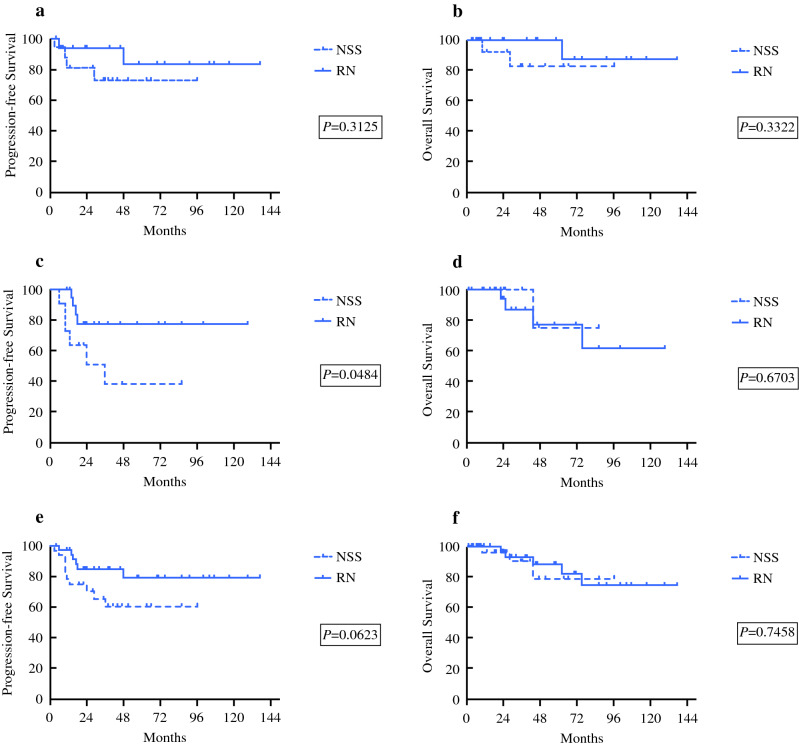
Survival analysis of Xp11.2 translocation renal cell carcinoma with comparison between radical nephrectomy and nephron-sparing surgery subgroups: **a** overall and **b** progression-free survival of patients with cT1a tumor; **c** overall and **d** progression-free survival of patients with cT1b tumor; and **e** overall and **f** progression-free survival of patients both with cT1a and cT1b tumor

To explore the feasibility of NSS in Xp11.2 translocation RCC, patients were divided into two subgroups according to surgical method: in the RN subgroup, no significant differences were observed in PFS or OS between Xp11.2 translocation RCCs and conventional RCCs (Supplementary Material 2), whereas in the NSS subgroup, the PFS and OS of Xp11.2 translocation RCCs were significantly poorer than those of conventional RCCs (Supplementary Material 3). Among patients with Xp11.2 translocation RCC at cT1b stage, those who underwent RN had more favorable PFS than those who underwent NSS. However, there was no significant difference either in PFS or OS between surgical methods among patients with Xp11.2 translocation RCC at cT1a stage and all cT1 stage (Fig. 2). Cox proportional hazards analysis showed that surgical method failed to be accepted as a risk factor in the cT1a and the cT1b groups (Supplementary Material 4, 5).

## Discussion

The first case of Xp11.2 translocation RCC was reported in 1986 6,16 but the tumor was not described as a clinicopathological entity until 2001 and 2002.^17^,^18^ Recently, Xp11.2 translocation RCC has attracted increasing attention, and hundreds of cases have been reported. Among these, children and adults under 45 years of age were the most frequently affected. The incidence rate of Xp11.2 translocation RCC was one-third among pediatric patients with RCC and 15% among adult patients with RCC under 45 years of age.^19^,^20^ Due to the aggressive biological behavior and younger age at onset, many cases in early literature were found to be locally invasive at their primary diagnosis and thus underwent RN.^6^,^7^ With developments in the field of medicine, a growing number of small renal masses (including Xp11.2 translocation RCCs) were checked out, and a considerable number of these underwent NSS. However, literature regarding oncologic outcomes after NSS for Xp11.2 translocation RCCs is scarce, especially with respect to the adult population. To the best of our knowledge, the present study is the first to systematically compare the anatomic location, features of pathological invasion, and prognosis of Xp11.2 translocation RCCs and conventional RCCs at cT1a and cT1b stages.

With increasing size of tumor, the likelihood of local invasion, such as invasion into renal sinus, renal vein or its tributaries, capsule, and perinephric fat, increases sharply.^21^^–^23 According to the study by Bonsib et al.,^23^ sinus invasion rate was 13% among ccRCCs with diameters smaller than 4 cm, 75% among those with diameters of 4.1–7 cm, and 97% among tumors larger than 7 cm. Besides, renal sinus involvement was associated with tumor histology, and its incidence was higher in ccRCC than in pRCC and chRCC.^23^ A considerable extent of local invasion was undetectable before the surgery, and patients subsequently underwent NSS.^24^,^25^ Our study revealed a higher local invasion rate in Xp11.2 translocation RCC, especially in patients with cT1b tumor. Altogether, 36.1% of the Xp11.2 translocation RCC at cT1a stage was undefined when compared with conventional RCC (12.4%). These results are consistent with the results reported by Cheng et al.,^26^ who reported that the incidence of pseudocapsule formation in Xp11.2 RCC was 63.6%.

Notably, lymphatic metastasis was common in Xp11.2 translocation RCC. This was observed in half of the patients with Xp11.2 translocation RCC at cT1b stage. A study by Ellis et al.,^6^ which is one of the biggest studies associated with regional lymph node involvement in Xp11.2 translocation RCC, reported that regional lymph node metastasis was observed in 24/32 (75%) of the ASPSCR1-TFE3 carcinomas and in 5/14 (35.7%) of the PRCC-TFE3 carcinomas. Taking all ASPSCR1-TFE3 and PRCC-TFE3 carcinomas into consideration, 7/16 (43.8%) T1a tumors and 6/12 (50.0%) T1b tumors showed regional lymph node involvement, which is consistent with our data. However, 11 out of 13 patients presenting with N1M0 disease remained disease-free in the short-term follow-up. Multivariate analysis showed that regional lymph node involvement did not portray a grim prognosis. Similar results were observed in the study by Galler et al.,^27^ wherein the majority of the patients with pediatric node-positive Xp11.2 translocation RCC survived without undergoing lymph node dissection. The authors proposed that “second look” lymph node dissections were not required for pediatric patients who did not undergo lymphadenectomy in the first procedure. However, it is unclear whether lymph node dissection was necessary for the treatment of Xp11.2 translocation RCC.

Pelvicalyceal invasion is another feature that needs attention. A high percentage of pelvicalyceal invasion in Xp11.2 translocation RCC is consistent with the common clinical presentation of gross hematuria.^20^,^28^ Due to the hollow structure of the renal pelvis, pelvicalyceal invasion is easier for localized RCC originating from the marginal parenchyma surrounding the renal pelvis.^29^,^30^ The anatomic features of endophytic growth, proximity to the collecting system, and central location relative to the polar lines observed in our study might explain the tendency of pelvicalyceal invasion in the Xp11.2 translocation RCC group. RCCs with pelvicalyceal invasion were possibly mistaken for transitional cell carcinoma, even after contrast-enhanced CT, CT urography, or magnetic resonance imaging (MRI).^31^,^32^ Four cases of Xp11.2 translocation RCC invading the renal pelvis in the present study initially mimicked transitional cell carcinoma and underwent ureteronephrectomy including cuff resection of the bladder wall. In the present study, none of the Xp11.2 translocation RCCs extended into the ureter or the bladder, which was different from the creeping feature of clear cell subtype observed in the dominant renal mass.^30^,^33^

A detailed understanding of the renal surgical anatomy is necessary while evaluating the feasibility of elective NSS. To date, the RENAL score designed by Kutikov et al. 15 is the most commonly used anatomical score system. The RENAL score has been revealed to be associated with histological features and aggressiveness.^34^,^35^ Renal lesions with low RENAL scores are usually associated with more indolent RCCs or benign histology.^36^,^37^ In addition, RENAL scores can predict postoperative recurrence and are negatively associated with OS.^38^,^39^ Our results showed that the “N” and the “L” categories of Xp11.2 translocation RCCs at cT1a stage tended to get a score of 3. However, the scores of conventional RCCs showed a relatively uniform distribution. Among patients with cT1b tumors, the “E” and the “N” scores of Xp11.2 translocation RCCs were higher than those of conventional RCCs. Thus, the anatomic location of Xp11.2 translocation RCC tended to be more central, which led to a higher total RENAL score. Therefore, the central location of Xp11.2 translocation RCCs made NSS difficult to perform, and RN was the surgical method of choice.

To date, only eight adult Xp11.2 translocation RCCs at pT1N0M0 stage (seven pT1a and one pT1b) have undergone NSS and maintained a stable course during a median follow-up of 13 and 37 months, respectively.^40^,^41^ In addition, 13 pediatric Xp11.2 translocation RCCs that underwent NSS have been reported. In the study by Ramphal,^42^ four pediatric Xp11.2 translocation RCCs underwent NSS and showed stable course with a median follow-up of 75 months. In the study by Liu et al.,^43^ nine children with tumor diameters less than 7 cm underwent NSS and achieved excellent outcomes, implying that NSS is an alternative treatment for pediatric Xp11.2 translocation RCCs measuring less than 7 cm. Unlike the indolent feature of pediatric cases, our results showed that, in adult Xp11.2 translocation RCCs, NSS achieved inferior oncologic outcomes than RN, even though the multivariate analysis of PFS did not identify surgical method as a risk factor. Nevertheless, NSS is an alternative for adult Xp11.2 translocation RCCs at cT1a stage.

As percutaneous puncture biopsy is not routinely performed for renal masses, Xp11.2 translocation RCCs are usually diagnosed postoperatively. In fact, Xp11.2 translocation RCC shares distinctive radiological features with conventional RCC, which could provide clues for the diagnosis of this rare tumor.^44^^–^47 Typical dynamic CT features of Xp11.2 translocation RCC include cystic-solid renal masses with circular calcifications in the unenhanced phase and “slow-in and delay-out” pattern in the contrast-enhanced phases.^44^,^45^ MRI findings have revealed that Xp11.2 translocation RCCs are isointense on T1-weighted imaging, heterogeneously hypointense on T2-weighted imaging, and slightly hyperintense on diffusion-weighted imaging.^46^ On contrast-enhanced ultrasound, Xp11.2 translocation RCCs showed obvious hypoenhancement with irregular nonenhanced regions in the early phase and the delayed phases.^47^ Thus, radiologic imaging could be applied to differentiate Xp11.2 translocation RCC from conventional RCC. A risk-scoring system for preoperative diagnosis of adult Xp11.2 translocation RCC combining imaging with epidemiological characteristics is in progress. Reasonable preoperative planning could help optimize the operative techniques and improve the outcomes of Xp11.2 translocation RCC.

The present study has some limitations. Even though our study had the largest number of patients in the NSS group compared with previous studies, the number was still insufficient. The retrospective and multicenter design of our research led to many inherent biases, especially the selection bias. The low overall incidence of this type of RCC was the primary reason for these limitations. Further prospective studies with a greater number of NSS patients are required to confirm our findings.

In conclusion, our initial experience of a multicenter study in the Chinese population identified the central location of Xp11.2 translocation RCC. For cT1a tumors, the oncologic outcomes of NSS were comparable to those of RN. However, NSS is not recommended for cT1b tumors due to the possibility of increased risk of postoperative recurrence and metastasis. The efficacy and the feasibility of NSS in Xp11.2 translocation RCC warrant large-scale studies and long-term follow-up.

## Electronic Supplementary Material

Below is the link to the electronic supplementary material.

Supplementary material 1 (DOCX 453 kb)
