# Supplementary material for: Nephron-Sparing Surgery for Adult Xp11.2 Translocation Renal Cell Carcinoma at Clinical T1 Stage: A Multicenter Study in China
Source: Ann Surg Oncol. 2020 Jul 6;28(2):1238–46. doi: 10.1245/s10434-020-08813-y (PMC7801353; doi:10.1245/s10434-020-08813-y)
Supplement: Supplementary file 1 — Supplementary material 1 (DOCX 453 kb) [file 10434_2020_8813_MOESM1_ESM.docx]

**Supplementary Material 1. Number of cases per institution**

| Centers | Xp11.2 translation RCC | Conventional RCC |
| --- | --- | --- |
| Nanjing Drum Tower Hospital | 49 | 195 |
| Jiangsu Province Hospital, | 11 | 40 |
| Jiangsu Cancer Hospital | 6 | 25 |
| Zhongda Hospital Southeast University | 2 | 7 |
| Nanjing First Hospital | 2 | 6 |


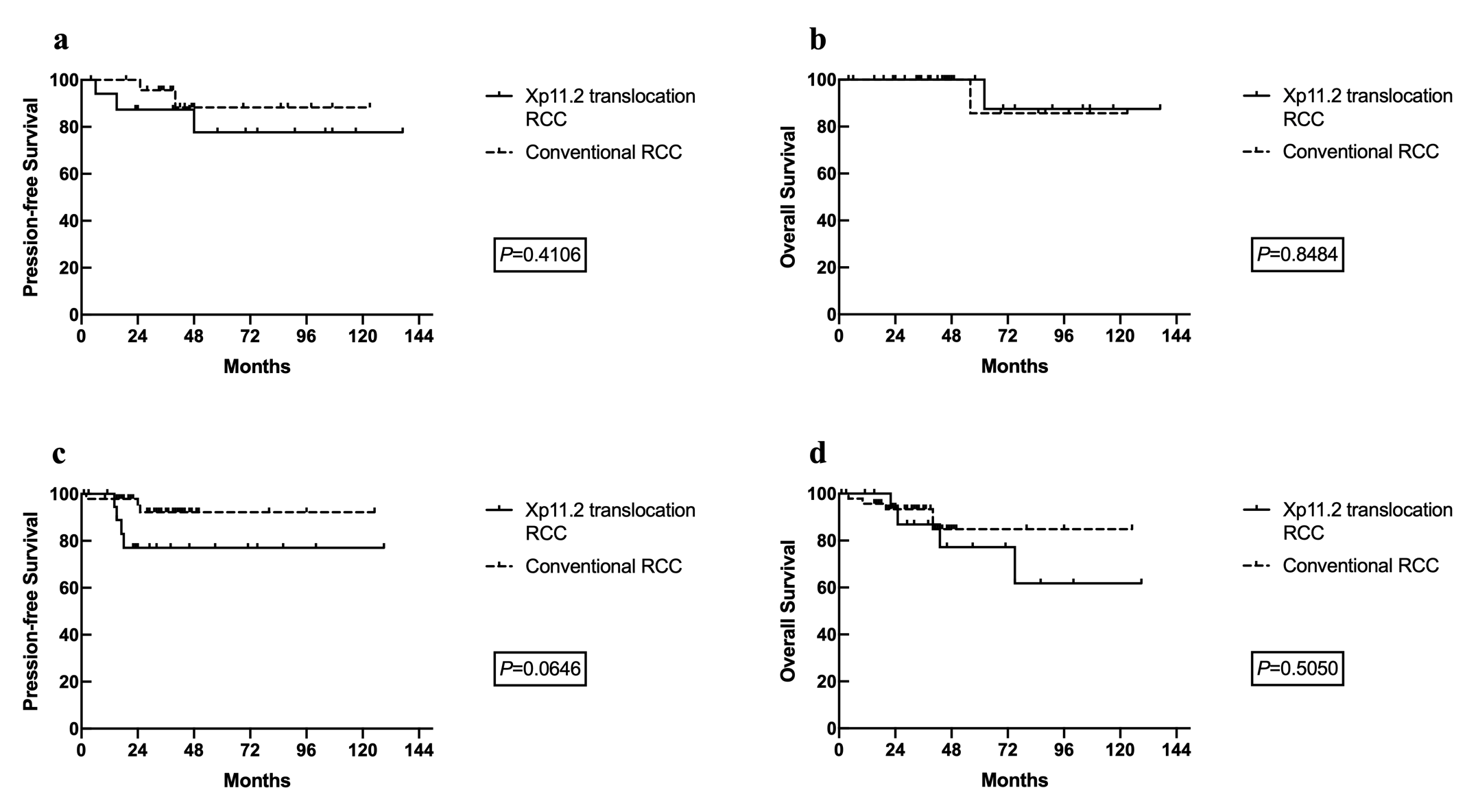


Supplementary Material 2. Survival analysis of radical nephrectomy subgroup with comparison between Xp11.2 translocation and conventional renal cell carcinoma

(a) and (b): Overall survival and progression-free survival of patients with cT1a tumor

(c) and (d): Overall survival and progression-free survival of patients with cT1b tumor


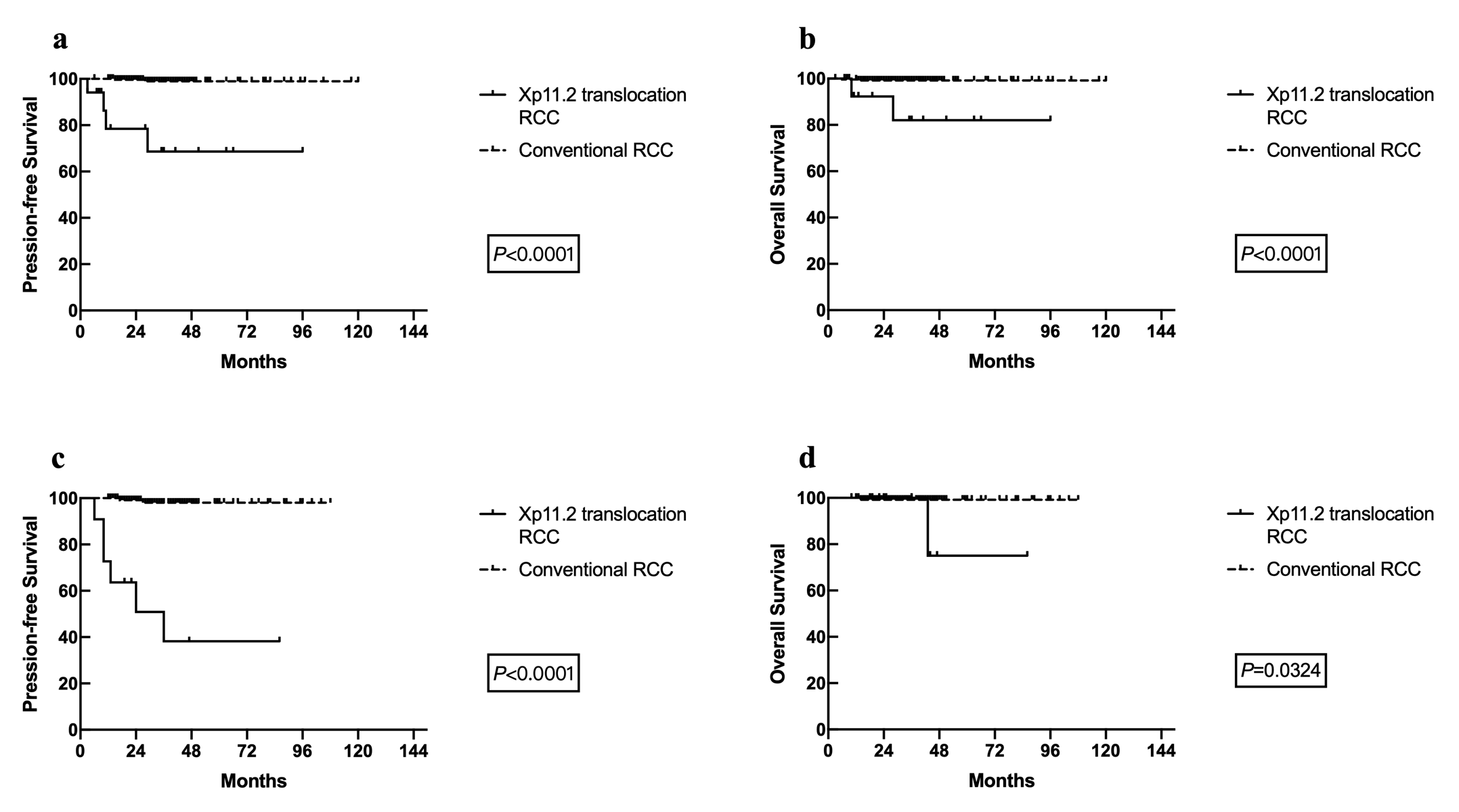


Supplementary Material 3. Survival analysis of the nephron-sparing surgery subgroup with comparison between Xp11.2 translocation and conventional renal cell carcinoma

(a) and (b): Overall survival and progression-free survival of patients with cT1a tumor

(c) and (d): Overall survival and progression-free survival of patients with cT1b tumor

**Supplementary Material 4. Cox proportional hazards analysis of clinicopathological factors to predict progression-free survival of Xp11.2 translocation renal cell carcinoma at clinical T1a stage**

NSS: nephron-sparing surgery; RN: radical nephrectomy

| Variables | Univariate | | Multivariate | |
| --- | --- | --- | --- | --- |
|  | HR (95 % CI) | P value | HR (95 % CI) | P value |
| Age (mean ± SD) | 1.041 (0.994-1.089) | 0.087 | - | - |
| Surgical margin (Negative VS. Positive) | 1.28 (0.285-5.741) | 0.747 | - | - |
| Fuhrman grade  (I-II VS. III-IV)3d | 2.792 (0.538-14.484) | 0.222 | - | - |
| Necrosis or Sarcomatous  (Without VS. With) | 5.657 (1.259-25.418) | 0.024 | 6.935 (0.028-2421.82) | 0.674 |
| Tumor Stage  (Local VS. Invasion^*^) | 10.177 (1.923-53.861) | 0.006 | 39.353 (0.129-4558.232) | 0.732 |
| Surgery (RN VS. NSS) | 1.818 (0.403-8.207) | 0.437 | 3.539 (0.179-69.794) | 0.406 |

Invasion* including breakthrough of renal capsule, undefined border, adrenal invasion, pelvicalyceal invasion, venous cancer thrombus, renal sinus invasion or lymphatic metastasis that identified by pathology.

**Supplementary Material 5. Cox proportional hazards analysis of clinicopathological factors to predict progression-free survival of Xp11.2 translocation renal cell carcinoma at clinical T1b stage**

| Variables | Univariate | | Multivariate | |
| --- | --- | --- | --- | --- |
|  | HR (95 % CI) | P value | HR (95 % CI) | P value |
| Age (mean ± SD) | 1.074 (1.023-1.126) | 0.004 | 1.503(0.065-34.654) | 0.799 |
| Surgical margin (Negative VS. Positive) | 3.607 (1.004-12.962) | 0.049 | 0.24(0.018-3.243) | 0.283 |
| Fuhrman grade  (I-II VS. III-IV) | 1.312 (0.367-4.693) | 0.676 | - | - |
| Necrosis or Sarcomatous  (Without VS. With) | 2.01 (0.564-7.158) | 0.281 | - | - |
| Tumor Stage  (Local VS. Invasion^*^) | 2.462 (0.706-8.582) | 0.157 | - | - |
| Surgery (RN VS. NSS) | 4.453 (0.527-17.384) | 0.276 | 2.77(0.092-83.783) | 0.558 |

NSS: nephron-sparing surgery; RN: radical nephrectomy

Invasion* including breakthrough of renal capsule, undefined border, adrenal invasion, pelvicalyceal invasion, venous cancer thrombus, renal sinus invasion or lymphatic metastasis that identified by pathology.
